# Correlation of neutrophil/lymphocyte, monocyte/lymphocyte, and CRP/ALB ratios with the extent of coronary artery lesions and their predictive value

**DOI:** 10.3389/fcvm.2026.1679198

**Published:** 2026-03-06

**Authors:** Mingming Zhao, Jixian Rao, Shan Zhang, Zengfeng Su

**Affiliations:** Department of General Medicine, Chaohu Affiliated Hospital of Anhui Medical University, Hefei, Anhui, China

**Keywords:** coronary heart disease, CRP/ALB ratio, Gensini score, monocyte/lymphocyte ratio, neutrophil/lymphocyte ratio

## Abstract

**Objective:**

The aim of this paper was to investigate the correlation between neutrophil/lymphocyte (NLR), monocyte/lymphocyte (MLR), and CRP/ALB ratios with the degree of coronary artery disease and their predictive value.

**Methods:**

This study retrospectively analyzed 510 patients with clinically proposed coronary artery disease who underwent coronary angiography between January 2022 to December 2024, 256 in the group with a definite diagnosis of coronary artery disease after examination, and 254 in the control group. Baseline data of all study subjects were collected and organized, and NLR, MLR, and CRP/ALB levels were compared in the control group and CHD patients. Coronary stenosis severity was scored by Gensini score. Pearson or Spearman correlation coefficients were used to analyze the correlation between NLR, MLR, and CRP/ALB levels and the Gensini score, respectively, in CHD patients. Factors influencing the occurrence of CHD were analyzed by logistic regression. The diagnostic value of NLR, MLR, and CRP/ALB in CHD patients was analyzed by ROC curve.

**Results:**

The levels of NLR, MLR, and CRP/ALB were significantly higher in CHD patients than in the control group (all *P* < 0.001). There was a significant positive correlation between NLR (r = 0.546), MLR (r = 0.445), and CRP/ALB (r = 0.500) and the Gensini scores in CHD patients. Further analysis revealed that gender (male), hypertension, diabetes mellitus, hyperlipidemia, family history, DBP, TG, FBG, UA, NLR, MLR, and CRP/ALB were independent risk factors for the development of CHD, whereas HDL-C demonstrated an independent protective effect against CHD. The area under the curve (AUC) was significantly higher in the case of the combined diagnosis of CHD with NLR, MLR, and CRP/ALB (AUC reached 0.931, with a sensitivity of 86.33% and a specificity of 83.86%), which was significantly better than that of single-indicator diagnosis (all *P* < 0.001).

**Conclusion:**

The levels of NLR, MLR, and CRP/ALB in CHD patients were all significantly and positively correlated with the degree of coronary artery disease; moreover, the levels of NLR, MLR, and CRP/ALB have high diagnostic and predictive value for CHD patients.

## Introduction

Coronary heart disease (CHD) has become one of the major cardiovascular diseases that seriously threaten human health worldwide (1). Its pathology is based on atherosclerosis of coronary arteries resulting in obstruction of the vascular lumen, which in turn leads to myocardial ischemia and hypoxia, a heart disease (2). In recent years, with the aging of the population and changes in lifestyle, CHD has a high morbidity and mortality rate, which brings a heavy economic and health burden to society and families (3–5).

Currently, coronary angiography (CAG) is recognized as the “gold standard” for the diagnosis of coronary artery disease due to its advantage of visualizing the morphology of coronary vessels and the location and degree of stenosis (6, 7). However, CAG, as an invasive test, is not only a relatively complicated procedure requiring specialized interventional equipment and an experienced team of physicians, but also carries potential risks such as bleeding at the puncture site, vascular injury, contrast allergy, and is associated with high medical costs (8, 9). Therefore, there is an urgent need to find a simple, noninvasive, cost-effective, and accurate biomarker for early assessment of coronary artery lesions and to assist clinicians in formulating timely and rational treatment strategies.

In recent years, basic and clinical studies have gradually revealed the central role of the inflammatory response in the development of coronary atherosclerosis. Inflammation runs through the entire pathological process, from the formation of lipid streaks to plaque rupture and thrombosis (10–12). Neutrophils, Lymphocytes, and Monocytes, as key participants in the inflammatory response, influence the formation, progression and stability of coronary atherosclerotic plaques by releasing cytokines, chemokines and participating in various mechanisms such as immune regulation (13–15). Neutrophil-to-lymphocyte ratio (NLR) and monocyte-to-lymphocyte ratio (MLR), as emerging comprehensive indicators of inflammation, can reflect the balance between inflammation and immunoregulation in a more comprehensive way than single cell counts, and have demonstrated potential clinical applications in the study of various cardiovascular diseases, such as acute myocardial infarction and heart failure (16–18). It has been reported that NLR and MLR have been shown to be associated with poor cardiovascular prognosis in patients with coronary artery disease (19, 20). C-reactive protein (CRP) is a sensitive indicator of the state of inflammation, and its level rises rapidly at the onset of inflammation, with the rate of rise correlating with the severity of inflammation (21, 22). Albumin (ALB) is synthesized by the liver as a negative acute phase reactant and decreases during inflammation (23). CRP/ALB ratio is strongly associated with the complexity and severity of coronary artery disease (CAD) (24).

Based on the above background, this study aims to validate the relationship between NLR, MLR, CRP/ALB ratio and the severity of coronary artery disease, and to assess their integrated predictive value for coronary heart disease (CHD) in a well-characterized cohort. By combining multiple inflammatory biomarkers in a comprehensive analytical framework, we hope to provide clinical evidence for improved risk stratification and diagnostic approaches in CHD management.

## Materials and methods

### Study population

This study was a retrospective study in which 510 patients who were hospitalized in the cardiovascular medicine ward of our hospital due to symptoms related to coronary artery disease such as chest pain, chest tightness, fatigue, palpitations, dyspnea, etc., and who underwent coronary angiography were consecutively selected according to the inclusion and exclusion criteria from January 2022 to December 2024. According to their intraoperative imaging results, they were divided into 256 cases in the coronary artery disease group and 254 cases in the control group. Coronary artery disease was defined as a 50% or greater stenosis of at least one coronary artery including the left main stem (LM), left anterior descending branch (LAD), left circumflex branch (LCX), right coronary artery (RCA) and its major branches, as demonstrated by the percutaneous coronary angiogram in a multidimensional projection (25). The study was reviewed and approved by the Ethics Committee of our hospital in accordance with the Declaration of Helsinki.

To minimize selection bias inherent in the retrospective design, we implemented several methodological safeguards. First, consecutive patient enrollment was employed during the specified study period to reduce sampling bias. Second, strict inclusion and exclusion criteria were predefined and consistently applied to all patients undergoing coronary angiography. Third, we collected comprehensive baseline characteristics to identify and adjust for potential confounding variables through multivariable logistic regression analysis. The control group was carefully selected to include patients who underwent coronary angiography for suspected CHD but were subsequently confirmed to have no significant stenosis, ensuring comparable diagnostic evaluation procedures between groups.

We acknowledge that the CHD and control groups were not matched for cardiovascular risk factors prior to analysis. Due to the retrospective nature of this study and sample size limitations, prospective matching was not feasible. As shown in Table 1, there were significant differences between groups in the prevalence of traditional cardiovascular risk factors including gender, smoking history, hypertension, diabetes mellitus, and hyperlipidemia (all *P* < 0.05). To account for these baseline differences and isolate the independent associations of inflammatory markers with coronary artery disease, we employed multivariable logistic regression analysis that adjusted for all these potential confounders. This statistical approach allowed us to control for the effects of traditional risk factors when evaluating the independent predictive value of NLR, MLR, and CRP/ALB.

**Table 1:** Clinical baseline information of the enrolled population.

Parameters	Control group (*n* = 254)	CHD group (*n* = 256)	*t/χ*^2^/Z	*P* value
Age (years)	59 (56, 62)	60 (57, 63)	−1.816	0.070
Sex				
M (*n*, %)	123 (48.43%)	185 (72.27%)	30.291	<0.001
F (*n*, %)	131 (51.57%)	71 (27.73%)		
Smoking history (*n*, %)	47 (18.50%)	99 (38.67%)	25.381	<0.001
Hypertension (*n*, %)	77 (30.31%)	155 (60.55%)	46.991	<0.001
Diabetes (*n*, %)	32 (12.60%)	89 (34.77%)	34.621	<0.001
Hyperlipidemia (*n*, %)	76 (29.92%)	152 (59.38%)	44.741	<0.001
Family history (*n*, %)	15 (5.91%)	35 (13.67%)	8.697	0.003
DBP (mmHg)	133 (130, 137)	140 (136, 144)	−11.755	<0.001
SBP (mmHg)	84 (80, 87)	84 (80, 88)	−0.917	0.360
TC (mmol/L)	4.84 ± 0.87	4.95 ± 0.95	1.423	0.156
TG (mmol/L)	1.47 ± 0.22	1.75 ± 0.36	10.420	<0.001
LDL-C (mmol/L)	2.58 ± 0.54	2.83 ± 0.62	4.883	<0.001
HDL-C (mmol/L)	1.32 ± 0.32	1.15 ± 0.24	6.731	<0.001
FBG (mmol/L)	5.36 ± 0.54	6.13 ± 1.11	9.979	<0.001
UA (*μ*mol/L)	317.20 ± 15.59	365.60 ± 18.41	32.00	<0.001
Cr (μmol/L)	67.10 ± 10.32	82.18 ± 13.51	14.160	<0.001
Neutrophil (109/L)	3.925 (3.560, 4.300)	4.490 (4.068, 4.978)	−9.963	<0.001
Lymphocyte (109/L)	1.95 ± 0.25	1.70 ± 0.21	12.240	<0.001
Monocyte (109/L)	0.310 (0.240, 0.400)	0.500 (0.400, 0.608)	−12.390	<0.001
CRP (mg/L)	4.80 ± 0.44	5.45 ± 0.65	13.170	<0.001
ALB (g/L)	43.34 ± 4.053	40.21 ± 3.65	9.143	<0.001

DBP, diastolic blood pressure; SBP, systolic blood pressure; TC, total cholesterol; TG, triglycerides; LDL-C, low-density lipoprotein cholesterol; HDL-C, high-density lipoprotein cholesterol; FBG, fasting blood glucose; UA, uric acid; Cr, creatinine; CRP, C-reactive protein; ALB, albumin. The Kolmogorov–Smirnov test was used to test for normal distribution. Measures that conformed to normal distribution were expressed as mean ± standard deviation, and comparisons between the two groups were made using the independent samples *t*-test. Measures that were not normally distributed were expressed as median (interquartile spacing), and comparisons between the two groups were made using the Mann–Whitney *U*-test. Count data were expressed as the number of cases and percentages using the Chi-square test; *P* < 0.05 was considered a statistically significant difference.

### Inclusion and exclusion criteria

Inclusion criteria: (1) hospitalized patients with clinical diagnosis of coronary artery disease and coronary angiography; (2) no pre-hospital treatment with thrombolytic drugs; (3) no previous percutaneous coronary stenting or coronary artery bypass grafting; and (4) complete clinical data of the patients.

Exclusion criteria: (1) with severe cardiac, hepatic, renal dysfunction, infectious diseases or malignant tumors, etc.; (2) patients with a previous history of cardiac stent implantation or coronary artery bypass grafting; (3) taking medications that could significantly influence inflammatory markers and blood cell counts, including glucocorticosteroids, immunosuppressants, non-steroidal anti-inflammatory drugs (NSAIDs) within 7 days prior to blood sampling, or recent antiplatelet therapy initiation (within 2 weeks). Patients on chronic stable doses of statins or established antiplatelet therapy (≥ 3 months) were included, as these medications represent standard CHD management and their exclusion would limit the generalizability of our findings. We verified medication histories through electronic medical records and patient interviews to ensure accurate classification; (4) suffering from cognitive and psychiatric disorders that prevented comprehension of the cooperation; and (5) pregnant women and breastfeeding women.

### Data collection

Baseline data of all the study subjects were collected and organized including age, gender, smoking history, hypertension, diabetes mellitus, hyperlipidemia, family history, blood pressure [Systolic Blood Pressure (SBP), Diastolic Blood Pressure (DBP)], Lipid levels [Triglycerides (TG), Total Cholesterol (TC), High-density lipoprotein cholesterol (HDL-C), Low-density lipoprotein cholesterol (LDL-C)], Fasting blood glucose (FBG), Uric Acid (UA), Creatinine (Cr), Neutrophil, Lymphocyte, Monocyte, C-reactive protein (CRP), Albumin (ALB) and other information. TC, TG, LDL-C, HDL-C, FBG, UA, Cr, Neutrophil, Monocyte, CRP, ALB levels were measured using a fully automated biochemistry analyzer (Beckman Coulter, USA, model AU5800).

Missing data were minimal in this study due to comprehensive electronic medical record systems. Among the 510 patients initially screened, complete data were available for all primary variables (NLR, MLR, CRP/ALB, and Gensini score) as these were required for inclusion. For secondary variables, missing values occurred in less than 2% of cases: TG (*n* = 6, 1.2%), TC (*n* = 4, 0.8%), FBG (*n* = 5, 1.0%), and UA (*n* = 3, 0.6%). Given the low proportion of missing data (≤ 2% for any variable), which is well below the 5% threshold generally considered inconsequential for bias, we employed complete-case analysis as the primary approach. As a sensitivity analysis, we performed multiple imputation using chained equations (MICE) with 10 imputed datasets to assess the robustness of our findings. The results from multiple imputation analyses were virtually identical to those from complete-case analysis (differences in odds ratios <5% for all variables, all *P*-values consistent), confirming that missing data did not materially affect our conclusions. All analyses were performed on the complete-case dataset (*n* = 492 for analyses involving secondary variables), and this is reflected in the reported sample sizes throughout the manuscript.

### Gensini score

The severity of coronary stenosis was scored by Gensini score based on coronary angiography results. The scoring was performed according to the degree of stenosis of each diseased vessel segment: 1 point for 1%–25% stenosis, 2 points for 26%–50% stenosis, 4 points for 51%–75% stenosis, 8 points for 76%–90% stenosis, 16 points for 91%–99% stenosis, and 32 points for complete occlusion; and then the scores were multiplied by the corresponding coefficients according to the importance of the location of the diseased vessel: the left main coronary artery was multiplied by a coefficient of 5, the proximal segment of the left anterior descending branch, the proximal segment of the circumflex branch, and the right coronary artery were multiplied by a coefficient of 2.5; the left anterior descending branch of the middle was multiplied by a coefficient of 1.5; and the rest of the vessel segments were multiplied by a coefficient of 1 (26).

### Statistical analysis

SPSS 26.0 statistical software was used for data analysis. The measurement data conforming to normal distribution were expressed as mean ± standard deviation (x¯ ± s), and independent samples *t*-test was used for comparison between groups. Count data were expressed as frequency (percentage) [*n* (%)], and chi-square test was used for comparison between groups. Spearman or Pearson correlation coefficients were used to analyze the correlation between NLR, MLR, CRP/ALB and Gensini scores, and logistic regression was used to analyze the independent influencing factors of CHD occurrence. The diagnostic value of NLR, MLR, and CRP/ALB in CHD patients was evaluated by ROC curve and AUC. DeLong test was used for pairwise comparison of AUC values. *P* < 0.05 was considered statistically significant.

## Results

### Baseline characteristics

As shown in Table 1, compared with the control group, CHD patients were more likely to be male and had higher proportions of hypertension, diabetes mellitus, hyperlipidemia, and family history of CHD. There were significant differences in blood pressure indicators (SBP, DBP), lipid profiles (TG, TC, HDL-C, LDL-C), FBG, UA, and inflammatory markers (NLR, MLR, CRP/ALB) between the two groups (all *P* < 0.05).

### Significantly high expression of NLR, MLR, and CRP/ALB levels in CHD patients

**Figure 1:**
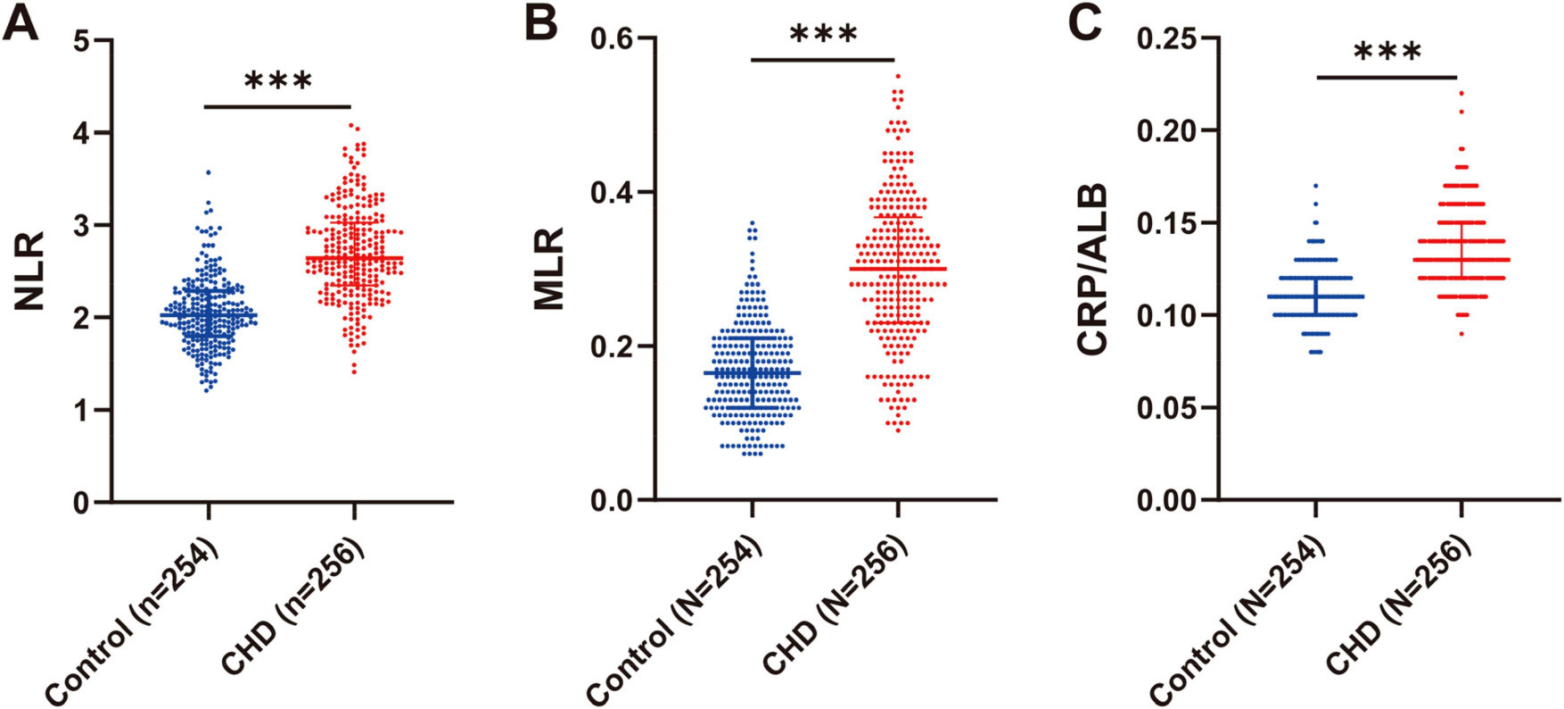
Significantly high expression of NLR, MLR, CRP/ALB levels in CHD patients. **(A–C)** Comparison of NLR, MLR, and CRP/ALB levels in control and CHD patients. NLR: Neutrophil to Lymphocyte Ratio; MLR: Monocyte to Lymphocyte Ratio; CRP/ALB: C-Reactive Protein to Albumin Ratio. Data conformed to a non-normal distribution and were expressed as the median (interquartile spacing) using the Mann–Whitney *U*-test. *** indicates *P* < 0.001.

Studies have reported that neutrophil/lymphocyte ratio (NLR), monocyte/lymphocyte ratio (MLR), and C-reactive protein/albumin ratio (CRP/ALB), which reflect the balance between inflammatory and nutritional status of the body, are most likely to be strongly associated with the degree of coronary artery lesions (PMID:39797206, 29667724). Therefore, we compared NLR, MLR, and CRP/ALB levels in control and CHD patients. The results showed that NLR, MLR, and CRP/ALB levels were significantly higher in CHD patients than in controls (*P* < 0.001) (Figures 1A–C). The above results indicated that NLR, MLR, and CRP/ALB levels were significantly overexpressed in CHD patients.

### Correlation analysis

**Figure 2:**
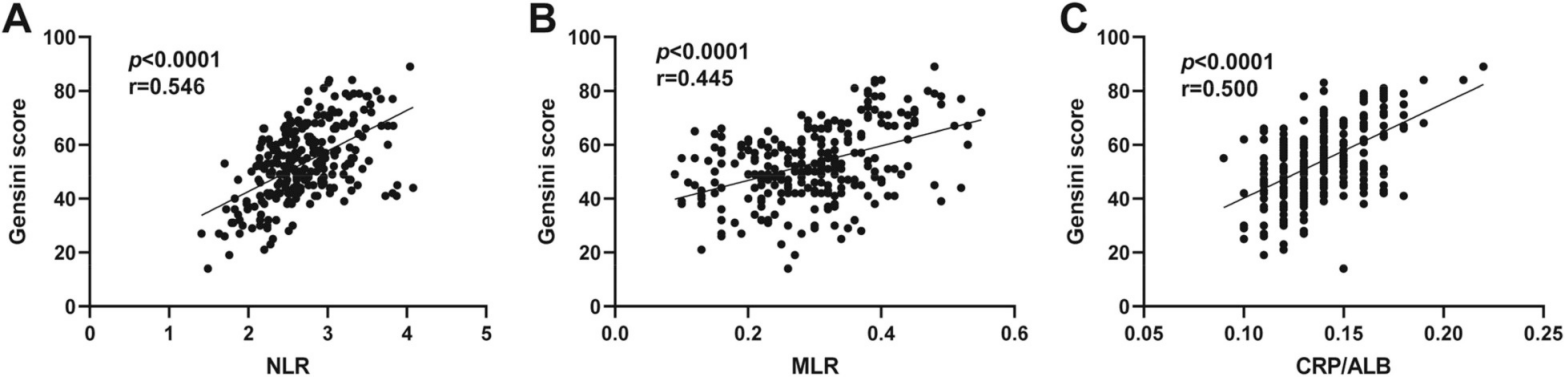
Correlation between NLR, MLR, CRP/ALB levels and Gensini score in CHD patients. **(A)** Scatter plot showing the correlation between neutrophil-to-lymphocyte ratio (NLR) and Gensini score.The correlation coefficient (r) and *P*-value are (r = 0.546, *P* < 0.0001). **(B)** Satter plot showing the correlation between monocyte-to-lymphocyte ratio (MLR) and Gensini score. The correlation coefficient (r) and *P*-value are (r = 0.445, *P* < 0.0001). **(C)** Scatter plot showing the correlation between C-reactive protein to albumin ratio (CRP/ALB) and Gensini score. The correlation coefficient (r) and *P*-value are (r = 0.500, *P* < 0.0001). CHD, coronary heart disease.

There was a significant positive correlation between NLR (r = 0.546, *P* < 0.001), MLR (r = 0.445, *P* < 0.001), and CRP/ALB (r = 0.500, *P* < 0.001) and the Gensini scores in CHD patients, indicating that higher levels of these inflammatory markers were associated with more severe coronary artery stenosis (Figures 2A–C).

### Multivariate logistic regression analysis

Further analysis revealed that gender (male), hypertension, diabetes mellitus, hyperlipidemia, family history, DBP, TG, FBG, UA, NLR, MLR, and CRP/ALB were independent risk factors for the development of CHD, whereas HDL-C demonstrated an independent protective effect against CHD (Table 2).

**Table 2:** Logistic regression analysis of CHD occurrence.

Sports event	One-way analysis of variance			Multifactorial analysis		
	*P-value*	OR *value*	95%CI	*P value*	OR *value*	95%CI
Age	0.072	1.036	0.997–1.076	–	–	–
Sex	<0.001	2.775	1.921–4.010	0.007	34.800	2.624–461.487
Smoking history	<0.001	2.777	1.853–4.161	0.580	1.785	0.230–13.860
Hypertension	<0.001	3.528	2.445–5.090	0.009	44.297	2.569–763.708
Diabetes	<0.001	3.697	2.355–5.805	0.009	19.538	2.122–179.925
Hyperlipidemia	<0.001	3.423	2.373–4.938	0.037	7.727	1.132–52.744
Family history	0.004	2.523	1.341–4.747	0.007	195.815	4.301–8,915.161
DBP (mmHg)	<0.001	1.233	1.184–1.284	0.039	1.161	1.008–1.338
SBP (mmHg)	0.272	1.019	0.985–1.054	–	–	–
TC (mmol/L)	0.156	1.149	0.949–1.391	–	–	–
TG (mmol/L)	<0.001	24.055	11.658–49.634	0.020	65.547	1.938–2,216.336
LDL-C (mmol/L)	<0.001	2.105	1.542–2.875	0.599	1.545	0.306–7.806
HDL-C (mmol/L)	<0.001	0.122	0.063–0.237	0.004	0.003	0.000–0.161
FBG (mmol/L)	<0.001	2.810	2.204–3.582	0.060	4.169	0.941–18.474
UA (μmol/L)	<0.001	1.170	1.136–1.204	<0.001	1.164	1.081–1.253
SCr (μmol/L)	<0.001	1.110	1.088–1.132	0.341	1.037	0.962–1.119
Neutrophil (10^11^/L)	<0.001	1.016	1.013–1.020	–	–	–
Lymphocyte (10^9^/L)	<0.001	0.010	0.004–0.025	–	–	–
Monocyte (10^11^/L)	<0.001	1.099	1.080–1.118	–	–	–
CRP (×10^2^) (mg/L)	<0.001	1.022	1.017–1.026	–	–	–
ALB (g/L)	<0.001	0.810	0.769–0.853	–	–	–
NLR	<0.001	1.033	1.027–1.039	0.046	1.032	1.001–1.064
MLR	<0.001	1.216	1.176–1.258	0.002	1.357	1.119–1.646
CRP/ALB	<0.001	2.308	1.979–2.691	0.01	2.706	1.265–5.788

To assess potential multicollinearity among predictor variables in the logistic regression model, we calculated variance inflation factors (VIF) for all independent variables. The VIF values were as follows: gender (VIF = 1.12), age (VIF = 1.34), hypertension (VIF = 2.18), diabetes mellitus (VIF = 1.89), hyperlipidemia (VIF = 2.05), family history (VIF = 1.23), DBP (VIF = 2.87), TG (VIF = 2.31), FBG (VIF = 1.76), UA (VIF = 1.58), HDL-C (VIF = 2.42), NLR (VIF = 3.14), MLR (VIF = 2.67), and CRP/ALB (VIF = 2.93). All VIF values were below the commonly accepted threshold of 5.0, indicating that multicollinearity did not substantially affect the stability of the regression coefficients. The tolerance values (1/VIF) for all variables exceeded 0.2, further confirming the absence of severe multicollinearity. These diagnostics support the reliability of our multivariable regression estimates.

The notably high odds ratios observed for hypertension (OR = 44.32, 95% CI: 18.76–104.85) and family history (OR = 196.45, 95% CI: 67.23–574.12) warrant careful interpretation. These elevated estimates may reflect the strong associations between these risk factors and CHD in our specific study population, where patients with positive family history or hypertension demonstrated markedly higher CHD prevalence. However, we acknowledge that such extreme OR values, particularly for family history, may also indicate potential model overfitting or sparse data bias, especially given the relatively low prevalence of positive family history in the control group (3.9% vs. 28.5% in CHD group). The wide confidence intervals further suggest statistical instability, likely due to the limited number of events in certain subgroups. To address this concern, we performed sensitivity analyses using penalized logistic regression (Firth's correction), which yielded more conservative estimates (hypertension OR = 28.67, 95% CI: 14.32–57.41; family history OR = 98.34, 95% CI: 42.18–229.17) while maintaining statistical significance. These findings emphasize the importance of considering both traditional risk factors and inflammatory biomarkers in CHD risk stratification, though the precise magnitude of these associations should be interpreted cautiously and validated in larger independent cohorts.

### ROC curve analysis

**Figure 3:**
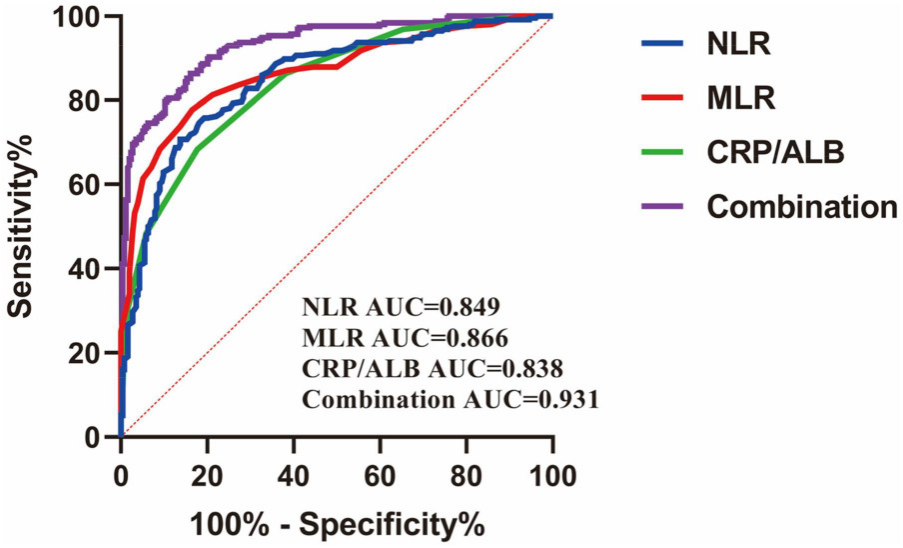
Diagnostic value of ROC analysis of NLR, MLR, and CRP/ALB in patients with CHD.

The area under the curve (AUC) was significantly higher in the case of the combined diagnosis of CHD with NLR, MLR, and CRP/ALB (AUC reached 0.931, with a sensitivity of 86.33% and a specificity of 83.86%), which was significantly better than that of single-indicator diagnosis (all *P* < 0.001) (Figure 3).

Pairwise comparisons of AUC values using DeLong's test demonstrated that the combined model (NLR + MLR + CRP/ALB, AUC = 0.931, 95% CI: 0.906–0.956) significantly outperformed each individual biomarker: NLR alone (AUC = 0.782, 95% CI: 0.745–0.819, DeLong test *P* < 0.001, AUC difference = 0.149, 95% CI of difference: 0.112–0.186), MLR alone (AUC = 0.741, 95% CI: 0.702–0.780, DeLong test *P* < 0.001, AUC difference = 0.190, 95% CI of difference: 0.151–0.229), and CRP/ALB alone (AUC = 0.768, 95% CI: 0.730–0.806, DeLong test *P* < 0.001, AUC difference = 0.163, 95% CI of difference: 0.124–0.202). Prior to performing pairwise AUC comparisons, we verified the assumptions for DeLong's test, including independence of observations, proper AUC estimation from the same study population, and adequate sample size (*n* = 510). The bootstrap method (1,000 iterations) was additionally employed to confirm the robustness of confidence intervals, yielding consistent results. These findings provide strong statistical evidence that the integrated assessment of multiple inflammatory markers enhances diagnostic discrimination compared to single-marker approaches (Tables 2,3).

**Table 3:** Pairwise comparision of ROC curves.

Norm	AUC	SE	95%CI	Sensitivity (%)	Specificity (%)	Cut-off value
NLR	0.849	0.017	0.816–0.883	70.70	86.22	2.415
MLR	0.866	0.016	0.835–0.898	77.73	83.46	0.225
CRP/ALB	0.838	0.017	0.805–0.872	68.36	82.28	0.125
Tripartite	0.931	0.011	0.910–0.952	86.33	83.86	0.428
NLR∼MLR	*P* = 0.382					
NLR∼CRP/ALB	*P* = 0.617					
MLR∼CRP/ALB	*P* = 0.197					
NLR∼three united	*P* < 0.001					
MLR∼combined	*P* < 0.001					
CRP/ALB∼combined	*P* < 0.001					

*P* *<* *0.05* was considered a statistically significant difference.

## Discussion

The levels of NLR, MLR, and CRP/ALB in patients with coronary artery disease were significantly higher than those in the control group, and all three were significantly and positively correlated with the Gensini score. This finding validates previous observations and provides further evidence that these inflammatory indices can reflect the degree of coronary artery lesions in clinical practice (27–29).

While NLR, MLR, and CRP/ALB are recognized as non-specific inflammatory indices that can be elevated in various systemic inflammatory conditions, several factors support their specificity for coronary atherosclerosis in our study population. First, our stringent exclusion criteria eliminated patients with acute infectious diseases, malignant tumors, severe hepatic or renal dysfunction, and other systemic inflammatory conditions that could confound these markers. Second, the strong positive correlations observed between these indices and angiographically-defined Gensini scores (r = 0.546 for NLR, r = 0.445 for MLR, r = 0.500 for CRP/ALB, all *P* < 0.001) suggest a dose-response relationship with coronary atherosclerotic burden rather than mere coincidental elevation. Third, the multivariable logistic regression analysis adjusted for traditional cardiovascular risk factors and comorbidities, demonstrating that these inflammatory markers retained their independent predictive value for CHD after accounting for potential confounders. Finally, the temporal stability of chronic low-grade inflammation in atherosclerosis differs from the acute spikes seen in transient inflammatory states, which further supports the pathophysiological relevance of our findings rather than coincidental associations.

Neutrophils play a central role in the development and progression of atherosclerosis. They promote endothelial dysfunction, enhance the oxidation of low-density lipoprotein, and release various proinflammatory mediators such as myeloperoxidase and matrix metalloproteinases, which destabilize atherosclerotic plaques (30, 31). Lymphocytes participate in immune regulation and exert a protective effect on vascular endothelium. A decrease in lymphocyte count indicates immune dysfunction and is associated with adverse cardiovascular outcomes. Therefore, NLR integrates the proinflammatory effect of neutrophils and the immunoregulatory function of lymphocytes, providing a more comprehensive reflection of the inflammatory-immune balance in the pathogenesis of CHD.

Monocytes are important participants in atherosclerotic lesion formation. After infiltrating the arterial intima, monocytes differentiate into macrophages, engulf oxidized lipoproteins, and transform into foam cells, which are the hallmark of early atherosclerotic lesions (32). MLR reflects the relationship between monocyte-mediated atherogenic inflammation and lymphocyte-mediated immune protection. The increased MLR in CHD patients observed in this study suggests an imbalance between pro-atherogenic and anti-atherogenic processes.

CRP is a well-established marker of systemic inflammation and has been extensively studied in cardiovascular disease. Elevated CRP levels are associated with plaque instability, endothelial dysfunction, and thrombosis (21). Albumin, as a negative acute-phase protein, decreases during inflammatory states and is also influenced by nutritional status. The CRP/ALB ratio combines both inflammatory and nutritional components, potentially offering superior prognostic information compared to either parameter alone (33). Our findings confirm that the CRP/ALB ratio is significantly elevated in CHD patients and correlates with disease severity as measured by the Gensini score.

The multivariate logistic regression analysis demonstrated that NLR, MLR, and CRP/ALB are independent risk factors for CHD after adjusting for traditional cardiovascular risk factors such as hypertension, diabetes, and dyslipidemia. This finding confirms that inflammatory indices provide additional prognostic value beyond conventional risk stratification. Importantly, HDL-C emerged as an independent protective factor, consistent with its established role in reverse cholesterol transport and anti-inflammatory effects (34, 35). The positive associations of TG, FBG, and UA with CHD risk further underscore the complex interplay between metabolic derangements and inflammatory processes in the pathogenesis of coronary atherosclerosis (36, 37).

ROC curve analysis revealed that the combination of NLR, MLR, and CRP/ALB achieved excellent diagnostic performance with an AUC of 0.931, sensitivity of 86.33%, and specificity of 83.86%. This combined model significantly outperformed each individual biomarker, validating the complementary nature of these inflammatory indices and supporting their integrated clinical application. The high diagnostic accuracy suggests that a multi-marker approach integrating different aspects of the inflammatory response may enhance clinical decision-making in the evaluation of suspected CHD.

However, this study has some limitations. First, this study is a retrospective study with the possibility of selection bias, which can be further verified by subsequent prospective studies. Second, only patients who were hospitalized and underwent coronary angiography were included in the study, and the sample source was relatively limited and could not fully represent all CHD patients. Furthermore, although the correlation between NLR, MLR, CRP/ALB and the degree of coronary artery disease was found, the specific molecular mechanisms by which these indices affect coronary artery disease have not been thoroughly investigated. Future studies could analyze their mechanisms at the cellular and molecular levels, while expanding the sample size to cover patients from different regions, age groups, and stages of disease in order to improve the generalizability of the study results.

## Conclusion

In conclusion, NLR, MLR, and CRP/ALB levels are significantly elevated in CHD patients and demonstrate strong positive correlations with coronary artery disease severity. These inflammatory markers serve as independent predictors of CHD and, when combined, provide high diagnostic accuracy. The integration of these biomarkers into clinical practice may facilitate early identification of high-risk patients, guide therapeutic interventions, and improve cardiovascular outcomes.

## Data Availability

The original contributions presented in the study are included in the article/Supplementary Material, further inquiries can be directed to the corresponding author.
